# Dr. Frank L. Tozier

**Published:** 1906-05

**Authors:** 


### OBITUARY.

Dr. Frank L. Tozier, of Batavia, N. Y., died at the German Deaconess's Hospital, April 23, 1906, after an operation for appendicitis two days previously, aged 35 years. He was the son of Dr. L. L. Tozier who, since 1875, has been the leader in his profession in Genesee County. Dr. Frank Tozier had already begun to occupy a leading place when stricken with his fatal illness. He received his preliminary education at the Batavia high school, and graduated in medicine at Bellevue Hospital Medical College in 1896. He began practice in association with his father, but later set out for himself, establishing an extensive and lucrative professional business. He was physician to the state school for the blind, having served three terms, and was just entering on the fourth when he died. He is survived by his wife, father, mother and sister, all residing at Batavia.

